# Is there an association between female gender and outcome in severe trauma? A multi-center analysis in the Netherlands

**DOI:** 10.1186/s13049-019-0589-3

**Published:** 2019-02-13

**Authors:** M. Pape, G. F. Giannakópoulos, W. P. Zuidema, E. S. M. de Lange-Klerk, E. J. Toor, M. J. R. Edwards, M. H. J. Verhofstad, T. N. Tromp, E. M. M. van Lieshout, F. W. Bloemers, L. M. G. Geeraedts

**Affiliations:** 1 Department of Trauma Surgery, VU University Medical Center, De Boelelaan 1117, P.O. Box 7057, 1007 MB Amsterdam, The Netherlands; 2 Department of Epidemiology and Biostatistics, VU University Medical Center, Amsterdam, The Netherlands; 3 Trauma Network North-West, VU University Medical Center, Amsterdam, The Netherlands; 4 Department of Trauma Surgery, Radboud University Medical Center, Nijmegen, The Netherlands; 5 Department of Trauma Surgery, Erasmus University Medical Center, Rotterdam, The Netherlands; 6 Trauma Research Unit, Department of Surgery, Erasmus University Medical Center, Rotterdam, The Netherlands

**Keywords:** Gender dimorphism, Trauma, Severe injury, Female gender, Mortality, ICU admission

## Abstract

**Introduction:**

Little evidence suggest that female gender is associated with a lower risk of mortality in severely injured patients, especially in premenopausal women. Previous clinical studies have shown contradictory results regarding protective effects of gender on outcome after severe trauma. The objective of this study was to determine the association between gender and outcome (mortality and Intensive Care Unit (ICU) admission) among severely injured patients in the Netherlands.

**Methods:**

A retrospective multicentre study was performed including all polytrauma patients (Injury Severity Score (ISS) ≥16) admitted to the ED of three level 1 trauma centres, between January 1st, 2006 and December 31st, 2014. Data on age, gender, mechanism of injury, ISS, Abbreviated Injury Scale (AIS), prehospital intubation, Revised Trauma Score (RTS), systolic blood pressure (SBP) and Glasgow Coma Scale (GCS) upon admission at the Emergency Department was collected from three Regional Trauma Registries. To determine whether gender was an independent predictor of mortality and ICU admission, logistic regression analysis was performed.

**Results:**

Among 6865 trauma patients, male patients had a significantly higher ISS compared to female patients (26.3 ± 10.2 vs 25.3 ± 9.7, *P =* < 0.0001). Blunt trauma was significantly more common in the female group (95.2% vs 92.3%, *P* = < 0.0001). Males aged 16- to 44-years had a significant higher in-hospital mortality rate (10.4% vs 13.4%, *P* = 0.046). ICU admission rate was significantly lower in females (49.3% vs 54.5%, *P* = < 0.0001). In the overall group, logistic regression did not show gender as an independent predictor for in-hospital mortality (OR 1.020 (95% CI 0.865–1.204), *P* = 0.811) or mortality within 24 h (OR 1.049 (95% CI 0.829–1.327), *P* = 0.693). However, male gender was associated with an increased likelihood for ICU admission in the overall group (OR 1.205 (95% CI 1.046–1.388), *P* = 0.010).

**Conclusion:**

The current study shows that in this population of severely injured patients, female sex is associated with a lower in-hospital mortality rate among those aged 16- to 44-years. Furthermore, female sex is independently associated with an overall decreased likelihood for ICU admission. More research is needed to examine the physiologic background of this protective effect of female sex in severe trauma.

## Introduction

The effect of gender in severely injured patients has been investigated for many years [1–7]. It is well known that severe trauma results in an inflammatory response with an aberrant production of cytokines by macrophages, resulting in suppression of the immune system [1]. This response is potentially gender dimorphic, for it has been known that cells of the immune system can synthesize sex steroids [1]. Several laboratory studies in rodents have demonstrated that female gender is protective after major trauma, hemorrhage and sepsis [2–4]. Despite the substantial experimental evidence, previous clinical studies have been unable to produce results resembling the laboratory findings [2, 6–8, 10–12, 14–16].

In a German study with 36,000 severely injured patients it was shown that gender alone offers no survival advantage after blunt trauma [5]. However, male gender was an independent negative predictor for morbidity [5]. In another study, no gender-based differences in mortality were shown, but it was concluded that age and ISS were the most important predictors of mortality [13]. In contrast, a large multicenter study demonstrated that severely injured men with an ISS equal to or above 25 and aged 50-years or younger, had a 27% increased likelihood of dying, compared with women [11]. Similarly, a retrospective Chinese cohort study showed that females had a lower risk of in-hospital mortality compared with males, especially among those 50-years or younger, with an ISS equal to 25 or higher [7]. These studies suggest that a potentially protective effect of female gender is most apparent during the reproductive phase [7, 11]. Furthermore, they speculate that minor trauma does not lead to immunologic changes and therefore gender-based differences might be more obvious in the severely injured [7].

In an attempt to further elucidate this ongoing controversy, a large retrospective multicenter study was performed to determine the association between gender and outcome, among severely injured patients, admitted at three Level 1 trauma centers in the Netherlands. We hypothesized that female polytrauma patients show decreased mortality rates and lower ICU admission rates.

## Patients and methods

### Study subjects

Patients aged 16 years or older and with an Injury Severity Score (ISS) ≥ 16 admitted to the Emergency Department (ED) between January 1st, 2006 and December 31st, 2014 were selected for inclusion. Patients who were dead upon arrival at the ED or presented after drowning, electrocution, or strangulation, were excluded. Patients were categorized into three age groups (i.e. 16–44, 45–54, and 55+), to consider the influence of hormonal status attributable to the premenopausal, perimenopausal, and postmenopausal phases. We varied this age threshold for defining the groups, ultimately leading to above mentioned groups. Because females between 16- and 44-years of age are considered hormonally active, a subgroup analysis was performed. Data were retrospectively collected from the regional trauma registries at three Level I trauma centers. These centers were the VU University Medical Center in Amsterdam, the Radboud University medical center in Nijmegen and the Erasmus university medical center in Rotterdam, all located in the Netherlands. The study was approved by the independent Medical Research Ethics Committees of all three hospitals.

### Study parameters

Demographic, injury and outcome parameters for each male and female patient were collected from the trauma registry. The injuries were classified by the Abbreviated Injury Scale (AIS) and the ISS [17, 18]. The Revised Trauma Score (RTS) was used to estimate the severity of the injury and dichotomized (12 or less yes/no). The ISS was used, both as a continuous variable, as well as a categorical variable (16–24, 25–50, and 51–75). Severe head injury was defined as a cranial AIS ≥ 3. Glasgow Coma Scale (GCS) at admission was also collected and dichotomized (eight or less yes/no). Severe thoracic and abdominal injury were defined as a thoracic or abdominal AIS ≥ 3, respectively. Mechanism of injury (MOI) was either blunt or penetrating. Hypotension was defined as a Systolic Blood Pressure (SBP) ≤ 89 mmHg at admission to the ED. Data on prehospital care provided by intubation, Physician Helicopter Emergency Medical Services (P-HEMS) assistance, ED intubation and hospital and ICU length of stay (LOS) was also collected. The primary study outcomes were: death within 24 h after admission and death until hospital discharge. Secondary outcomes were: rate of ICU admission and ICU LOS.

### Statistical analysis

For univariate assessment, independent students *t*- and chi-squared tests for continuous or categorical variables, respectively, were used on the overall and subgroup population. For skewed variables, median and range were given and a Mann-Whitney-U-test was performed. Backward stepwise logistic regression analyses on the effect of gender on outcome parameters were performed. Odds ratios (ORs) are reported with 95% confidence intervals (95% CI). In addition to gender, the regression models included the covariates age, ISS, year of admission, MOI, prehospital intubation, P-HEMS assistance rate, cranial, thoracic and abdominal AIS ≥ 3, RTS at the ED, and GCS and SBP on admission. *P*-values < 0.05 in two-sided tests were accepted as statistically significant. All analyses were done using IBM SPSS Statistics 23.0.

## Results

### Overall patient characteristics

A total of 6865 patients were admitted to three Level − 1 trauma centers from 2006 through 2014; 70.6% was male. Characteristics of the study population are shown in Table 1. Male patients were younger than female patients (47.7 ± 20.2 vs 55.4 ± 22.0, *P* < 0.0001). In the age groups 16- to 44-years and 45- to 54-years, males were significantly more represented (Table 2). The median ISS was 25 and 24 (*P* < 0.0001) for males and females, respectively. Severe head injury was more frequent in females (71.2% vs 63.2%, *P* < 0.0001). An AIS thorax ≥3 (46.1% vs 35.1%, *P* < 0.0001) and an AIS abdomen ≥3 (11.3% vs 9.5%, *P* = 0.032) were more frequent in males. Blunt trauma represented the dominant MOI for both sexes (92.3% vs 95.2%, *P* < 0.0001). Males were more frequently intubated at the scene (30.9% vs 27.7%, *P* = 0.009). P-HEMS assistance was significantly more frequent in males (38.1% vs 29.0%, *P* < 0.0001). Males had a higher rate of being intubated whilst in the ED (43.7% vs 39.3%, *P* = 0.003). Furthermore, hypotension at admission to the ED was also more frequent in males (6.8% vs 4.9%, *P* = 0.008).

**Table 1:** Patient characteristics by gender

	Total (*n* =6865)	Males (*n*=4851)	Females (*n*=2014)	*P*-value
Age (years) mean ± SD	49.9 ± 21.0	47.7 ± 20.2	55.4 ± 22.0	<0.0001
Age groups (%)				
16-44 years	2861 (41.7)	2212 (45.5)	652 (32.4)	<0.0001
45-54 years	1083 (15.8)	821 (16.9)	261 (13.0)	<0.0001
55+ years	2921 (42.5)	1818 (37.5)	1101 (54.7)	<0.0001
ISS median (range)	25.0(16-75)	25.0 (16-75)	24.0 (16-75)	<0.0001
ISS categorical (%)				
16-24	3417 (49.8)	2376 (49.0)	1041 (51.7)	0.044
25-50	3309 (48.2)	2369 (48.8)	940 (46.7)	0.108
51-75	139 (2.0)	106 (2.2)	33 (1.6)	0.171
Blunt mechanism of injury (%)	6396 (93.2)	4478 (92.3)	1918 (95.2)	<0.0001
AIS cranial ≥ 3 (%)	4502 (65.6)	3068 (63.2)	1434 (71.2)	<0.0001
AIS thorax ≥ 3 (%)	2944 (42.9)	2237 (46.1)	707 (35.1)	<0.0001
AIS abdomen ≥ 3 (%)	738 (10.8)	547 (11.3)	191 (9.5)	0.032
SBP ≤ 89 mmHg at admission (%)	429 (6.2)	330 (6.8)	99 (4.9)	0.008
GCS at admission median (range)	14.0 (3-15)	14.0 (3-15)	14.0 (3-15)	0.048
P-HEMS assistance (%)	2432 (35.4)	1847 (38.1)	585 (29.0)	<0.0001
Prehospital intubation (%)	2057 (30.0)	1500 (30.9)	557 (27.7)	0.009
Intubation at ED (%)	2912 (42.4)	2120 (43.7)	792 (39.3)	0.003

*AIS, Abbreviated Injury Scale; ED, Emergency Department; GCS, Glasgow Coma Scale; ISS, Injury Severity Score; P-HEMS, Physician staffed Helicopter Emergency Medical Service; SBP, Systolic Blood Pressure; SD, Standard Deviation*

**Table 2:** Patient characteristics by gender among those aged 16-44-years old

	Total (*n*=2861)	Males (*n*=2209)	Females (*n*=652)	*P*-value
Age (years) mean ± SD	29.0 ± 8.4	29.1 ± 8.4	28.6 ± 8.6	0.180
ISS median (range)	25.0 (16-75)	25.0 (16-75)	25.0 (16-75)	0.072
ISS categorical (%)				
16-24	1348 (47.1)	1024 (46.4)	324 (49.7)	0.134
25-50	1428 (49.9)	1118 (50.6)	310 (47.5)	0.169
51-75	85 (3.0)	67 (3.0)	18 (2.8)	0.719
Blunt mechanism of injury (%)	2580 (90.2)	1971 (89.2)	609 (93.4)	0.002
AIS cranial ≥ 3 (%)	1704 (59.6)	1313 (59.4)	391 (60.0)	0.808
AIS thorax ≥ 3 (%)	1455 (50.9)	1140 (51.6)	315 (48.3)	0.139
AIS abdomen ≥ 3 (%)	474 (16.6)	358 (16.2)	116 (17.8)	0.339
SBP ≤ 89 mmHg at admission (%)	201 (7.0)	165 (7.5)	36 (5.5)	0.217
GCS at admission median (range)	13.0 (3-15)	13.0 (3-15)	14.0 (3-15)	0.110
P-HEMS assistance (%)	1218 (42.6)	963 (43.6)	255 (39.1)	0.110
Prehospital intubation (%)	895 (31.3)	709 (32.1)	186 (28.5)	0.225
Intubation at ED (%)	1285 (44.9)	1012 (45.8)	273 (41.9)	0.184

*AIS, Abbreviated Injury Scale; ED, Emergency Department; GCS, Glasgow Coma Scale; ISS, Injury Severity Score; P-HEMS, Physician staffed Helicopter Emergency Medical Service; SBP, Systolic Blood Pressure; SD, Standard Deviation*

### Overall clinical outcomes

No differences for death until hospital discharge were found in the overall group (18.5% vs 17.5%, *P* = 0.379). No difference in 24-h mortality rate was found between females and males (6.9% vs 7.6%, *P* = 0.334) (Table 3). Females had a significantly lower ICU admission rate (49.3% vs 54.5%, *P* < 0.0001). The median ICU LOS was two days for males (range 0–184 days) and females (0–200 days), *P* < 0.0001).

**Table 3:** Clinical outcome comparison by gender

	Total (*n* =6865)	Males (*n*=4851)	Females (*n*=2014)	*P*-value
LOS (days) mean (range)	15.0 (0-220)	15.3 (0-202)	14.2 (0-220)	0.070
ICU stay (days) mean (range)	5.7 (0-200)	5.8 (0-184)	5.3 (0-200)	<0.0001
ICU admission rate (%)	3638 (53.0)	2646 (54.5)	992 (49.3)	<0.001
In-hospital mortality rate (%)	1223 (17.8)	851 (17.5)	372 (18.5)	0.379
24h mortality rate (%)	508 (7.4)	369 (7.6)	139 (6.9)	0.334

*ICU, Intensive Care Unit; LOS, Length of Stay*

### The overall association between 24-h mortality and gender

Backward logistic regression analysis did not show gender as independently associated with 24-h mortality (OR 1.049; 95% CI 0.829–1.327; *P* = 0.693; Table 4). However, age, P-HEMS assistance, blunt injury, ISS, AIS thorax and abdomen ≥3, hypotension at ED admission (SBP ≤ 89 mmHg), and prehospital intubation were identified as independent predictors for death. No statistically significant difference was found for gender when we varied the age threshold for defining the subgroups (OR 1.100, 95% CI 0.867–1.394; *P* = 0.433; Table 5).

**Table 4:** Clinical outcome comparison by gender among those aged 16-44-years old

	Total (*n*=2861)	Males (*n*=2209)	Females (*n*=652)	*P*-value
LOS (days) mean (range)	15.9 (0-220)	15.6 (0-201)	17.3 (0-220)	0.081
ICU stay (days) mean (range)	6.1 (0-200)	5.8 (0-114)	6.9 (0-200)	0.651
ICU admission rate (%)	1649 (57.6)	1275 (57.7)	374 (57.4)	0.458
In-hospital mortality rate (%)	364 (12.7)	296 (13.4)	68 (10.4)	0.046
24h mortality rate (%)	192 (6.7)	159 (7.2)	33 (5.1)	0.055

*ICU, Intensive Care Unit; LOS, Length of Stay*

**Table 5:** Logistic regression analysis of 24h mortality, in-hospital mortality and ICU admission on gender and other covariates in trauma patients (ISS≥16)

	24h mortality			In-hospital mortality			ICU admission		
Variable	OR	95% CI	*P* - value	OR	95% CI	*P* - value	OR	95% CI	*P* - value
Male Gender	1.049	0.829-1.327	0.693	1.020	0.865-1.204	0.811	1.205	1.046-1.388	0.010
Age, years									
16-44	REF		<0.0001^a^	REF		<0.0001^a^	REF		0.002^a^
45-54	0.972	0.688-1.373	0.872	1.232	0.961-1.580	0.100	0.906	0.749-1.096	0.311
55+	2.179	1.715-2.767	<0.0001	3.922	3.284-4.684	<0.0001	0.770	0.665-0.891	<0.0001
Blunt MOI	0.477	0.338-0.674	<0.0001	0.561	0.422-0.745	<0.0001	0.734	0.562-0.959	0.023
P-HEMS	1.321	1.042-1.675	0.021	1.212	1.021-1.439	0.028	1.234	1.057-1.441	0.008
Admission at ED, year									
2004-2006	1.548	0.800-2.992	0.194	1.315	0.826-2.094	0.249	0.724	0.492-1.066	0.102
2007-2009	2.214	1.615-3.035	<0.0001	1.533		<0.0001	1.014	0.832-1.235	0.893
2010-2012	1.441	1.070-1.940	0.016	1.224	1.230-1.909	0.049	0.615	0.523-0.724	<0.0001
2013-2014	REF		<0.0001^a^	REF	1.001-1.498	<0.0001^a^	REF		<0.0001^a^
ISS									
16-24	REF		<0.0001^a^	REF		<0.0001^a^	REF		<0.0001^a^
25-50	2.985	2.235-3.987	<0.0001	3.653	3.029-4.407	<0.0001	2.190	1.898-2.526	<0.0001
51-75	3.873	2.302-6.514	<0.0001	8.657	5.646-13.274	<0.0001	2.126	1.139-3.970	0.018
AIS Thorax ≥ 3	0.647	0.514-0.814	<0.0001	0.593	0.499-0.705	<0.0001	2.053	1.757-2.399	<0.0001
AIS Abdomen ≥ 3	1.646	1.202-2.255	0.002	1.405	1.088-1.813	0.009	1.776	1.406-2.245	<0.0001
Prehospital intubation	2.042	1.605-2.598	<0.0001	1.674	1.405-1.994	<0.0001	0.756	0.640-0.893	0.001
SBP ≤89 mmHg at ED	5.502	4.179-7.245	<0.0001	3.030	2.358-3.895	<0.0001	0.372	0.281-0.494	<0.0001
GCS ≤8 at ED	9.221	6.287-13.526	<0.0001	5.200	4.139-6.532	<0.0001	2.262	1.841-2.778	<0.0001
RTS≤12 at ED	1.034	0.667-2.670	0.880	1.352	1.034-1.767	0.027	3.460	2.844-4.209	<0.0001
AIS Head ≥ 3				1.479	1.196-1.829	<0.0001	1.155	0.977-1.365	0.091

^a^ *P* – value represents the overall significance across the categories

*AIS, Abbreviated Injury Scale; CI, Confidence Interval; ED, Emergency Department; GCS, Glasgow Coma Scale; ISS, Injury Severity Score; MOI, Mechanism of Injury; P-HEMS, Physician staffed Helicopter Emergency Services; OR, Odds ratio; SBP, Systolic Blood Pressure; RTS, Revised Trauma Score*

### The overall association between in-hospital mortality and gender

Backward logistic regression analysis did not show gender as independent risk factor for in-hospital mortality in the overall study population (OR 1.020; 95% CI 0.865–1.024; *P* = 0.811). The same variables that were identified as independent predictors for 24-h mortality, were also independent predictors for in-hospital mortality (Table 4). No statistically significant difference was found for gender when we varied the age threshold for defining the subgroups (OR 1.085; 95% CI 0.917–1.283; *P* = 0.341; Table 5).

### The overall association between ICU admission and gender

Backward logistic regression analysis showed that the male gender was independently associated with a 20.5% increased likelihood for ICU admission compared to the female gender (OR 1.205; 95% CI 1.05–1.39; *P* = 0.010; Table 4). A statistically significant difference was found for gender when we varied the age threshold for defining the subgroups (OR 1.192; 95% CI 1.035–1.374; *P* = 0.015; Table 5).

### Patient characteristics of those aged 16- to 44-years

Females and males were found to have comparable age, ISS, GCS, and AIS (Table 2). Females had a significantly higher rate of blunt injury (93.4% vs 89.2%, *P* = 0.002). The rates of hypotension, P-HEMS assistance and (prehospital) intubation were similar.

### Clinical outcomes in the 16- to 44 age group

Females did have significantly lower in-hospital mortality rates than to males (10.4% vs 13.4%, *P* = 0.046) (Table 6). No significant differences in 24-h mortality, ICU LOS and admission, or hospital LOS were found between men and women.

**Table 6:** Logistic regression analysis of 24h mortality, in-hospital mortality and ICU admission on gender and other covariates in trauma patients (ISS≥16) aged 16-44-years

	24h mortality			In-hospital mortality			ICU admission		
Variable	OR	95% CI	*P*- value	OR	95% CI	*P*- value	OR	95% CI	*P*- value
Male Gender	0.939	0.567-1.554	0.806	1.158	0.827-1.621	0.393	0.982	0.799-1.208	0.867
Blunt MOI	0.231	0.107-0.498	<0.0001	0.327	0.215-0.497	<0.0001	1.563	0.927-2.638	0.094
ISS									
16-24	REF		<0.0001^a^	REF		<0.0001^a^	REF		<0.0001^a^
25-50	6.546	3.447-12.432	<0.0001	3.769	2.527-5.621	<0.0001	3.463	2.769-4.332	<0.0001
51-75	8.090	2.153-30.399	0.002	7.858	4.105-15.041	<0.0001	9.550	4.370-20.868	<0.0001
Prehospital intubation				2.305	1.687-3.149	<0.0001	1.262	1.000-1.593	0.050
SBP ≤89 mmHg at ED	4.173	2.186-7.965	<0.0001	4.638	3.096-6.949	<0.0001	1.807	1.233-2.647	0.002
GCS ≤8 at ED	4.251	2.200-8.215	<0.0001	13.266	7.688-22.890	<0.0001	4.434	3.365-5.844	<0.0001
RTS≤12 at ED	3.293	1.340-8.094	0.009	0.868	0.476-1.585	0.646	1.615	1.167-2.234	0.004
AIS Head ≥ 3	1.795	0.978-3.296	0.059	2.189	1.491-3.215	<0.0001			
AIS Thorax ≥ 3	0.603	0.363-1.004	0.052	0.601	0.446-0.812	0.001	0.569	0.453-0.714	<0.0001
AIS Abdomen ≥ 3				1.565	1.076-2.276	0.019	1.532	1.006-2.333	0.047
Admission at ED, year									
2004-2006				1.247	0.524-2.968	0.617			
2007-2009				2.489	1.654-3.746	<0.0001			
2010-2012				1.775	1.209-2.605	0.003			
2013-2014				REF		<0.0001^a^			

^a^ *P* – value represents the overall significance across the categories

*AIS, Abbreviated Injury Scale; CI, Confidence Interval; ED, Emergency Department; GCS, Glasgow Coma Scale; ISS, Injury Severity Score; MOI, Mechanism of Injury; OR, Odds ratio; SBP, Systolic Blood Pressure; RTS, Revised Trauma Score*

### The association between 24-h mortality and gender in the 16- to 44 age group

Backward logistic regression analysis did not show gender independently associated with 24-h mortality (OR 0.939; 95% CI 0.567–1.554, *P* = 0.806). When we varied the age threshold (i.e. 16–40 age group), no statistically significant differences were found (Table 7). In this group, other variables were identified as independent predictors; blunt injury, ISS, SBP ≤ 89 mmHg, RTS ≤ 12, and AIS cranial and thorax ≥3 (Table 8.)

**Table 7:** Logistic regression analysis of <24h mortality, in-hospital mortality and ICU admission on gender and other covariates in trauma patients (ISS≥16)

	<24h mortality			In-hospital mortality			ICU admission		
Variable	OR	95% CI	*P* - value	OR	95% CI	*P* - value	OR	95% CI	*P* - value
Male Gender	1.100	0.867-1.394	0.433	1.085	0.917-1.283	0.341	1.192	1.035-1.374	0.015
Age, years									
16-40	REF	REF	<0.0001^a^	REF	REF	<0.0001^a^	REF	REF	<0.0001^a^
41-60	1.147	0.866-1.518	0.338	1.468	1.196-1.802	<0.0001	0.876	0.746-1.029	0.108
60+	2.784	2.142-3.619	<0.0001	5.336	4.381-6.499	<0.0001	0.708	0.603-0.832	<0.0001
Blunt MOI	0.462	0.327-0.654	<0.0001	0.539	0.405-0.717	<0.0001	0.739	0.566-0.965	0.027
P-HEMS	1.321	1.041-1.676	0.022	1.217	1.024-1.447	0.026	1.227	1.051-1.433	0.010
Admission at ED, year									
2004-2006	1.593	0.820-3.095	0.170	1.382	0.864-2.211	0.177	0.720	0.489-1.061	0.097
2007-2009	2.269	1.651-3.119	<0.0001	1.589	1.272-1.984	<0.0001	1.011	0.830-1.232	0.914
2010-2012	1.463	1.085-1.974	0.013	1.244	1.014-1.526	0.036	0.615	0.523-0.723	<0.0001
2013-2014	REF	REF	<0.0001^a^	REF	REF	<0.0001	REF	REF	<0.0001
ISS									
16-24	REF	REF	<0.0001^a^	REF	REF	<0.0001	REF	REF	<0.0001^a^
25-50	3.069	2.288-4.117	<0.0001	3.692	3.055-4.463	<0.0001	2.199	1.906-2.538	<0.0001
51-75	4.083	2.414-6.905	<0.0001	8.834	5.745-13.585	<0.0001	2.109	1.130-3.938	0.019
AIS Thorax ≥ 3	0.645	0.509-0.819	<0.0001	0.608	0.510-0.724	<0.0001	2.048	1.752-2.394	<0.0001
AIS Abdomen ≥ 3	1.667	1.207-2.304	0.002	1.492	1.154-1.929	0.002	1.756	1.389-2.220	<0.0001
Prehospital intubation	2.095	1.645-2.669	<0.0001	1.721	1.443-2.053	<0.0001	0.755	0.639-0.892	0.001
SBP ≤89 mmHg at ED	5.456	4.140-7.188	<0.0001	2.976	2.315-3.827	<0.0001	0.374	0.282-0.496	<0.0001
GCS ≤8 at ED	9.535	6.496-13.998	<0.0001	5.464	4.335-6.886	<0.0001	2.260	1.840-2.776	<0.0001
RTS≤12 at ED	1.029	0.663-1.597	0.897	1.357	1.036-1.777	0.027	3.464	2.848-4.215	<0.0001
AIS Head ≥ 3				1.461	1.180-1.810	0.001	1.163	0.984-1.374	0.077

*AIS, Abbreviated Injury Scale; CI, Confidence Interval; ED, Emergency Department; GCS, Glasgow Coma Scale; ISS, Injury Severity Score; MOI, Mechanism of Injury; P-HEMS, Physician staffed Helicopter Emergency Services; OR, Odds ratio; SBP, Systolic Blood Pressure; RTS, Revised Trauma Score*

^a^ *P* – value represents the overall significance across the categories

**Table 8:** Logistic regression analysis of <24h mortality, in-hospital mortality and ICU admission on gender and other covariates in trauma patients (ISS≥16) aged 16- to 40-years

	Early (<24h) mortality			In-hospital mortality			ICU admission		
Variable	OR	95% CI	*P*- value	OR	95% CI	*P*- value	OR	95% CI	*P*- value
Male Gender	1.498	0.903-2.484	0.117	1.224	0.847-1.767	0.282	1.057	0.810-1.379	0.682
Blunt MOI	2.377	1.425-3.964	0.001	3.196	2.055-4.968	<0.0001			
ISS									
16-24	REF	REF	0.009	REF	REF	<0.0001^a^	REF	REF	<0.0001^a^
25-50	2.234	1.314-3.798	0.003	3.408	2.217-5.239	<0.0001	2.256	1.754-2.902	<0.0001
51-75	2.610	1.168-5.834	0.019	6.834	3.404-13.722	<0.0001	1.432	0.628-3.265	0.393
Prehospital intubation	3.304	2.109-5.176	<0.0001	2.376	1.700-3.322	<0.0001	0.576	0.430-0.773	<0.0001
SBP ≤89 mmHg at ED	7.482	4.645-12.052	<0.0001	4.278	2.780-6.583	<0.0001	0.302	0.188-0.485	<0.0001
GCS ≤8 at ED	15.046	7.050-32.111	<0.0001	14.074	7.740-25.593	<0.0001	2.828	1.969-4.063	<0.0001
RTS≤12 at ED				0.842	0.436-1.625	0.608	4.390	3.089-6.238	<0.0001
AIS Head ≥ 3				0.495	0.328-0.747	0.001			
AIS Thorax ≥ 3	1.676	1.107-2.537	0.015	1.520	1.100-2.101	0.011	0.532	0.418-0.678	<0.0001
AIS Abdomen ≥ 3	0.589	0.365-0.949	0.030	0.593	0.399-0.881	0.010	0.527	0.382-0.729	<0.0001
Admission at ED, year									
2004-2006	0.768	0.190-3.098	0.711	1.101	0.425-2.852	0.843	0.711	0.371-1.362	0.304
2007-2009	3.543	1.957-6.414	<0.0001	2.790	1.788-4.355	<0.0001	0.863	0.606-1.230	0.415
2010-2012	1.522	0.848-2.732	0.160	1.749	1.145-2.674	0.010	0.473	0.352-0.635	<0.0001
2013-2014	REF	REF	<0.0001^a^	REF	REF	<0.0001^a^	REF	REF	<0.0001^a^

*AIS, Abbreviated Injury Scale; CI, Confidence Interval; ED, Emergency Department; GCS, Glasgow Coma Scale; ISS, Injury Severity Score; MOI, Mechanism of Injury; OR, Odds ratio; SBP, Systolic Blood Pressure; RTS, Revised Trauma Score*

^a^ *P* – value represents the overall significance across the categories

### The association between in-hospital mortality and gender in the 16- to 44 age group

Gender was not independently associated with in-hospital mortality in this age group (OR 1.158; 95% CI 0.827–1.621; *P* = 0.393; Table 8). When we varied the age threshold (i.e. 16–40 age group), no statistically significant differences were found (Table 7). Blunt injury, ISS, prehospital intubation, hypotension at ED admission (SBP ≤ 89 mmHg), GCS ≤ 8, admission year and AIS cranial, thorax and abdomen ≥3 were found independent predictors.

### The association between ICU admission and gender in the 16- to 44 age group

The male gender was not independently associated with ICU admission rate (OR 0.982; 95% CI 0.799–1.208; *P* = 0.867). When we varied the age threshold (i.e. 16–40 age group), no statistically significant differences were found (Table 7). ISS, prehospital intubation, hypotension at ED admission (SBP ≤ 89 mmHg), GCS ≤ 8, RTS ≤ 12, and AIS cranial, thorax and abdomen ≥3 were identified as independent predictors (Table 8).

## Discussion

There is an increasing amount of laboratory and clinical data, suggesting gender dimorphism in severely injured patients [1–7, 9].

This study is the first multicenter analysis performed on this subject in the Netherlands. The advantage of this retrospective study is the large sample size over an extended study period.

In this study, a statistically significant survival advantage was found in female patients age 16 to 44-years old. Overall, females had significantly lower ICU admission rates. Logistic regression analyses did not show gender as an independent predictor for early and in-hospital mortality in any subset population. However, the male gender was independently associated with a 20.5% increased likelihood for ICU admission. In this study, patients were divided in three age groups; 16- to 44- years old, 45- to 54- years old, and 55- years old or older. These categories were surrogates for the pre-, peri- and postmenopausal stages and were defined based on literature findings [19, 23]. As stated before, we varied the age thresholds for defining these groups. We found that varying these thresholds did not changed our results, therefore we continued using the previous stated groups (16- to 44- years old, 45- to 54- years old, and 55- years old or older). Women one year after the onset of menopause show a decline in estrogen levels, which may explain the absence of beneficial effects in women 55-years old or older in our population [1, 3, 15]. Considering postmenopausal women using hormone replacement therapy (HRT) a minority, it seems unlikely that this would have affected the results of the current study. To investigate the influence of aging, we excluded every patient who was 19 years or younger, or 61 or older in a subgroup analysis. The population that remained included 4132 patients. In-hospital mortality, mortality within 24 h and ICU admission, did not show a statistically significant difference. As described earlier, some studies found that severely injured men of 50- years or younger had a greater chance of not surviving (27%) than women of the same age [11]. This survival advantage for women of 50 years or younger was attributed to interactions between the immune and endocrine systems of both sexes, and not to a difference in MOI, severity or pattern [11]. Similarly, a meta-analysis of 19 studies found a survival advantage for women older than 50 years with a low to moderate injury (ISS ≤ 25). Interestingly, this advantage was not shown in severe trauma [33]. In a large retrospective cohort study it was demonstrated that female blunt trauma patients with an ISS ≥ 16 and relevant bleeding had lower rates of multiple organ failure (MOF) and sepsis [6]. After stratification by age group, these findings were most evident in groups of females at reproductive age (i.e., 16–44 years) [6]. They found no differences in the age group 55- to 64-years old. However, the possibility of late onset menopause or HRT in these women cannot be ruled out completely [6].

Another study stratified severely injured patients by MOI and age, assuming women of 50-years or older to be postmenopausal [8]. A major difference compared to the current study is that they excluded patients ages 41- to 50 years old because women in this age group were most likely to be in the perimenopausal stage of their menstrual cycle. The authors found a survival advantage for women aged below 40 years old and with an ISS between 16 and 24, although not statistically significant [8]. Logistic regression analyses also failed to identify gender as independently associated with mortality and are therefore consistent with our results.

Significant differences in mechanism and pattern of injury among male and female patients were found in our population. Females had more AIS ≥ 3 injuries to the head (71.2%) and males had more AIS ≥ 3 injuries to the chest (46.1%) and abdomen (11.3%). Blunt trauma represented the dominant mechanism of injury for both male and female patients (92.3% vs 95.2%, *P* < 0.0001). Male patients suffered more often from motorcycle accidents (7.3% vs 3.3%) and penetrating injuries (5.2% vs 2.8%). Penetrating trauma may differ from blunt by treatment and associated injuries. Since a small group suffered from penetrating injuries, the data reported would be unlikely to reveal a significant difference if these were analysed solely. Our study therefore did not further investigate the different trauma mechanisms (for instance whether it was a fall from height or a motor cycle accident) because it was not the main purpose of this article.

The current study also found that the male gender was an independent predictor of ICU admission after correction for several clinical important confounders. A probable explanation could be that some unknown confounders, such as hormonal status, were not included in the analysis. However, studies demonstrated that younger males (aged 45- years or younger) showed a significant higher frequency of MOF and mortality and also showed a longer ICU stay than females [24, 31]. An earlier study showed a significantly higher ICU admission rate for male surgical intensive care patients (64.2% vs 35.8%). Therefore they state that despite comparable age and surgical procedures, female patients may not be as critically ill as male patients [35, 36]. One clinical prospective cohort managed to measure sex steroids during ICU stay of severely injured patients and assess mortality [34]. They found a significantly elevated estradiol level in non-survivors, with the most severe injured patients having the highest levels [34]. Furthermore, after comparison with other parameters, the ability of estradiol to predict death was comparable with the ISS. One limitation of their study is that only a single estradiol level was drawn, 48 h after admission [34]. It is however, not known how quickly estrogens are elevated [34].

However, we also believe that whether or not the trauma patient is admitted to the ICU is influenced mostly by the physician on duty and cannot be solely be attributed to medical status such as injury severity or trauma mechanism. We therefore can only speculate that hormonal status may be considered a risk factor for ICU admission in severely injured patients.

Previous clinical studies, investigating gender-based differences in severely injured patients were often unable to produce results resembling the experimental data [2, 6–8, 10–12, 14–16, 30–32].

Experimental studies in rodents have demonstrated the female gender to be protective after severe injury and hemorrhage [1–3]. Other studies demonstrated that resistance to trauma or hemorrhage induced organ injury, varies during the menstrual cycle, with maximal protection during the proestrus stage, when estrogen levels are the highest [1, 3, 5]. However, when estrogen exceeded the physiological level in female rodents after trauma-hemorrhage, resembling pregnancy and burn injuries, the immune response was down-regulated [7–9, 20, 21].

These findings suggest that high end physiological levels of estrogen are considered beneficial on survival after trauma or hemorrhage [2, 3, 22, 29].

The retrospective nature of this study has some limitations. Since the retrospective trauma registry did not collect any data on reproductive cycle or hormonal status, we divided the patients in three subgroups, as alternates for hormonal status. Women younger than 55- years old were assumed to be pre- or perimenopausal and those aged 55- years or older to be postmenopausal. Another limitation is the lack of information on comorbidity and medication. Factors such as obesity, surgical and medical history, smoking habits, and the use of contraceptive or HRT are known to influence the actual hormonal status and could be possible confounders [8, 10, 23, 24].

We did not stratify the population based on the anatomical sites of injury (and their combinations), since this might lead to so many subgroups that sensitive analysis will not be possible. It is well known that trauma research is hampered by a heterogeneous population. We therefore decided to enter ISS and other surrogates for injury severity (i.e. AIS Cranial, Thorax and Abdomen) in our analysis to cope with the heterogeneity of the trauma patient population.

We are aware that there exist much larger registry studies with over 20,000 cases [5, 6, 8, 11, 14, 28]. Our study consist of 6865 cases. Our sample may be too small to detect a survival advantage for female trauma patients.

We attempted to use as much standardized definitions as possible to create a homogeneous study population with minimal variations. The Shock Index (SI), defined by the ratio of heart rate to systolic blood pressure, is the most commonly used clinical parameter to signal hemorrhagic shock [25–27]. However, the trauma registry lacked data on heart rate at admission for most patients. Therefore, shock was defined as a SBP ≤ 89 mmHg at admission, based on recent literature findings [8, 12, 16, 24, 26]. Using this definition may have underestimated the effect of hemorrhagic shock on mortality.

We conclude that in this population of severely injured patients, the female gender shows a lower mortality rate in patients aged 16- to 44- years. However, the male gender was independently associated with an increased likelihood for ICU admission. Gender was not independently associated with mortality, despite the large amount of experimental evidence.

Future research should focus on large, prospective multicentre observational studies, in which the actual hormonal status is adequately measured upon admission to the ED, hereby allowing to group patients more precisely by their cycle status and thus observe a clearer hormone-related relationship to their outcome.
